# Creation of Composite Aerogels Consisting of Activated Carbon and Nanocellulose Blended with Cross-Linked Biopolymers: Application as Ethylene Scavengers

**DOI:** 10.3390/polym16213081

**Published:** 2024-10-31

**Authors:** Asadullah, Kittaporn Ngiwngam, Jaejoon Han, Pornchai Rachtanapun, Rafael Auras, Thomas Karbowiak, Duangjai Noiwan, Masubon Thongngam, Wirongrong Tongdeesoontorn

**Affiliations:** 1 School of Agro-Industry, Mae Fah Luang University, 333 Moo 1 Tasud, Chiang Rai 57100, Thailand; asadullah.research@mfu.ac.th (A.); 6671401004@lamduan.mfu.ac.th (K.N.); 2 Research Center of Innovative Food Packaging and Biomaterials Unit, Mae Fah Luang University, 333 Moo 1 Tasud, Chiang Rai 57100, Thailand; 3 School of Chemical Engineering, Institute of Engineering, Suranaree University of Technology, 111 University Avenue, Nakhon Ratchasima 30000, Thailand; 4 Research Center of Chemical Engineering Department, Balochistan University of Informational Technology, Engineering and Management Sciences, Quetta 87300, Pakistan; 5 Department of Food Bioscience and Technology, College of Life Sciences and Biotechnology, Korea University, Seoul 02841, Republic of Korea; jjhan@korea.ac.kr; 6 Division of Packaging Technology, School of Agro-Industry, Faculty of Agro-Industry, Chiang Mai University, Chiang Mai 50100, Thailand; pornchai.r@cmu.ac.th; 7 The Cluster of Agro Bio-Circular-Green Industry (Agro BCG), Chiang Mai University, Chiang Mai 50100, Thailand; 8 Center of Excellence in Materials Science and Technology, Chiang Mai University, Chiang Mai 50200, Thailand; 9 School of Packaging, Michigan State University, 448 Wilson Rd, East Lansing, MI 48824, USA; 10 Institut Agro, INRAe, UMR PAM 1517, Université Bourgogne Franche-Comté, 1 Esplanade Erasme, 21000 Dijon, France; thomas.karbowiak@agrosupdijon.fr; 11 Department of Postharvest Technology, Faculty of Engineering and Agro-Industry, Maejo University, Chiang Mai 50290, Thailand; duangjai_nw@mju.ac.th; 12 Department of Food Science and Technology, Faculty of Agro-Industry, Kasetsart University, Bangkok 10900, Thailand; masubon.t@ku.th

**Keywords:** activated carbon, nanocellulose, aerogel, ethylene gas, biopolymer, macadamia nutshell

## Abstract

This study involved producing aerogels using activated carbon (AC) and nanocellulose (NC). Two distinct structured composites, AC composite aerogel (ACCA) and NC composite aerogel (NCCA), were developed by separately mixing AC and NC with identical proportions of cross-linked biopolymers: hydroxypropyl methylcellulose (HPMC), methylcellulose (MC), and chitosan (C). These aerogels were evaluated for their capability to adsorb ethylene gas through batch experiments, while the physical and chemical characteristics were thoroughly examined to determine their feasibility of removing ethylene. The resulting ACCA and NCCA aerogels exhibited low densities of 0.094 g cm^−3^ and 0.077 g cm^−3^, respectively, coupled with high porosity ranging between 95 and 96%. During the ethylene adsorption test, NCCA exhibited superior ethylene removal rates (~14.88–16.77 mL kg^−1^) compared to ACCA (~13.57–14.97 mL kg^−1^). Specifically, NCCA achieved a removal efficiency of 83.86% compared to 74.64% for ACCA. Kinetic model fitting yielded high R^2^ values ranging from 0.97 to 0.98 with the Lagergren kinetic model. These findings suggest the potential of composite aerogels to be incorporated into food packaging materials for dynamic ethylene capture, independent of environmental conditions, thereby providing promising routes for further development.

## 1. Introduction

Food packaging plays a critical role in the food processing industry by catering to the diverse needs of consumers. Hundreds and thousands of tons of food (fruits and vegetables) are spoiled due to an accelerated ripening process during transportation and storage [1]. Reducing food loss along production and supply chains and waste at the retail- and consumer-levels is an important and meaningful way to streamline food system costs, enhance food security, and contribute to environmental sustainability [1]. Ethylene, a plant growth regulator, speeds up the softening and ripening of fruits while they are transported and stored [2]. Ethylene triggers chlorophyll depletion, resulting in discoloration, and contributes to excessive softening of fruits, among other negative impacts, ultimately hastening deterioration and reducing post-harvest life [3]. Considering the worldwide urgency to reduce post-harvest losses of fruits and vegetables by 50% by 2030, as outlined in the United Nations Sustainable Development Goals, controlling ethylene in the post-harvest chain of such products is of utmost importance [4]. Therefore, proper post-harvest handling techniques are paramount to maintaining high-quality produce from harvesting until consumption. Different types of ethylene scavengers have been utilized to delay the ripening process of food, such as TiO_2_/SiO_2_ nanocomposite [5], chitosan/zeolite composite [4], activated carbon [6], metal–organic frameworks [7], and cellulose hydrogels [8]. However, a major problem with these materials is their environmental hazard due to the excessive use of toxic chemicals to synthesize them. Secondly, their efficiency is not up to the international standard once commercialized. Potassium permanganate (KMnO_4_) is a widely used commercial ethylene scavenger; however, some concerns remain due to its potential for health, environmental, and safety issues. Since potassium permanganate acts as an oxidizing agent, it releases oxygen both in neutral and alkaline environments. For instance, when ethylene reacts with alkaline potassium permanganate, it forms ethane-1,2-diol or ethylene glycol, and a visible color change takes place. Therefore, utilizing KMnO_4_ as an ethylene scavenger has drawbacks because it may eliminate desired aromas in fruit, and there is a risk of KMnO_4_ migrating directly into the product.

Recently, aerogels have gained much attention as food preservatives due to their convenience, high porosity, and enhanced surface properties [9,10]. The production process of aerogel involves solvent removal from gels while maintaining the network structure. Aerogel networks comprise bonded particles or nanoscale fibers, loosely arranged, leading to elevated levels of porosity (typically ranging from 94% to 99.99%) and exceptionally high specific surface areas (150 m^2^·g^−1^ and beyond). Additionally, bio-based aerogels have been developed using cellulose nanofibers sourced from coconut shells [11], jute [12], wheat straw [13], and other plants [14]. Nonetheless, cellulose-based aerogels exhibit certain limitations. For instance, the aerogel framework is sensitive to moisture, leading to a notable decrease in mechanical properties under a specific relative humidity (RH) range. Cellulosic materials typically contain dispersed hydroxyl groups on their molecules, rendering them susceptible to physical or chemical modification. These alterations can potentially impart new functionalities to cellulose aerogels, such as less hydrophilicity, enhanced mechanical properties, electromagnetic capabilities, and bioactivity, among others. This expanded functionality could broaden their range of applications.

Apart from being environmentally friendly and safe, the ethylene scavenger should also contain antioxidant and antimicrobial properties to enhance its efficiency. When packaged, food not only deteriorates due to ethylene formation but is also affected by the growth of several bacteria and molds. Utilizing natural bio-based polymers presents an economically and environmentally sustainable strategy for entering the food packaging industry. Chitosan-based aerogels are ideal for sustainable packaging due to their antibacterial properties, large surface area enhancing heat insulation, and compatibility with diverse materials for hybrid composites. Nevertheless, pure chitosan aerogel exhibits mechanical strength, thermal stability, and acid resistance deficiencies. Researchers have incorporated chitosan (C) with other polymers to address these limitations [15,16]. However, their effectiveness for diverse applications remains constrained due to certain factors. Efforts are underway to enhance bio-based packaging by exploring nanostructured biopolymer configurations and hybrid solutions. When making a composite, introducing biopolymer could reduce the aerogel’s volume shrinkage to allow for a larger volume. The investigation of the packaging industry relating to the potential of bio-based composite aerogels for sustainable packaging needs to be improved. Recent studies have demonstrated the effect of AC and NC for the preparation of hydrogels and aerogels used for different applications [17]. In a recent work [18], the authors prepared carbon-based aerogels that showed excellent CO_2_ adsorption capacity (93.98 cm^3^⋅g^−1^ at 0 °C) because of the largest pore volume (1.55 cm^3^⋅g^−1^). However, post-carbonization of biopolymer-based aerogels may decrease the product yield and could affect the antioxidant properties of the aerogels. Alternately, AC and NC obtained from biomass are biodegradable and nontoxic when used in food packaging material. However, their potential to be used in food packaging materials is limited. Although bio-based aerogels are increasingly acknowledged as sustainable packaging materials, a notable research gap exists in fully exploring their potential in this field. The complete spectrum of opportunities and challenges related to incorporating bio-based aerogels into food packaging materials needs to be thoroughly examined to bridge this gap.

This study highlights the existing gaps and obstacles in aerogel research about food applications and explores potential future niche markets for food-grade aerogels. Therefore, to develop biodegradable and efficient aerogels with high surface area and porosity, pre-carbonized AC and NC were cross-linked with biopolymers. Finally, sol–gel and freeze-drying methods were applied to fabricate the aerogels. The prepared aerogels were analyzed for their efficacy as ethylene scavengers, and the kinetic models were employed to discern the adsorption mechanism involved in the process.

## 2. Materials and Methods

### 2.1. Materials

Waste macadamia nutshells (MNSs) of the Integrifolia species were provided by a local macadamia processing factory in Chiang Rai, northern Thailand. Chitosan (C) of analytical grade with food grade certification, with a degree of acetylation greater than 90% and a molar mass of about 100 kDa, was purchased from Krungthepchemi Co., Ltd., Bangkok, Thailand. All the other organic precursor materials, such as hydroxypropyl methylcellulose (MC) and methylcellulose (MC), were purchased from Krungthepchemi Co., Ltd., Bangkok, Thailand. All the reagents and chemicals were purchased from Sigma Aldrich Chemicals, Pvt, Ltd., Bangkok, Thailand. All the additional chemicals and reagents, including H_3_PO_4_, H_2_SO_4_, and HCl, were obtained in analytical-grade quality from Ajax Finechem, Nonthaburi, Thailand and Loba Chemie, Bangkok, Thailand.

### 2.2. Methods

#### 2.2.1. Preparation of Macadamia Nutshell Activated Carbon (AC)

The MNSs underwent purification with distilled water followed by a 24 h drying period at 105 °C. Around 100 g of dried MNSs were then introduced into a furnace F62730 (Thermolyne, Lilienthal, Germany) for carbonization, sourced from the School of Science, Mae Fah Luang University, Chiang Rai, Thailand. This furnace was equipped with an external resistance heater and an internal thermocouple sensor for temperature monitoring. The carbonization was initiated with a gradual temperature increase to 500 °C, which was maintained for 3 h. Following this, the treated sample was transferred into a tube reactor with a measuring size of 6 cm in diameter and 50 cm in length, positioned within a horizontal tube furnace STF55433C-1 model (Thermo Fisher Scientific, Bangkok, Thailand) and connected to a nitrogen line. Inside the pyrolysis reactor, the heating rate was controlled at 3 °C min^−1^ until reaching 800 °C, where it was held for 2 h under a continuous nitrogen flow of 20 mL min^−1^ [19]. After cooling to room temperature, the resulting solid material was pulverized into a powder using a ball mill Pulverisette 5 (Fritsch, Idar-Oberstein, Germany), washed thoroughly with DI water, and dried. After size distribution to 100 µm, the resulting activated carbon was stored in a zip bag.

#### 2.2.2. Preparation of Macadamia Nutshell Nanocellulose (NC)

Ultra-sonication offers an alternative method for dispersing cellulose fibers through the hydrodynamic forces of ultrasound. This method produces mechanical oscillating power, resulting in the creation, expansion, and subsequent collapse of microscopic gas bubbles as liquid molecules absorb ultrasonic energy. Therefore, to avoid higher energy consumption and access to the use of chemicals, we adopted an ultrasonication technique with mild use of chemicals for NC production [20]. Briefly, 100 g of previously cleaned and dried MNSs were crushed and ground using a jaw crusher and a laboratory hammer mill HM-5 (Retsch, Haan, Germany). The ground MNSs were first soaked overnight in distilled water and then washed with warm distilled water several times to remove unwanted impurities. Consequently, a 5 g MNS sample was added to an Erlenmeyer flask with 250 mL of NaOH 5% *w*/*v* in a ratio of MNS weight to NaOH volume of 1:50 (g:mL). The mixture was ultra-sonicated for 2 h at 80 °C. Moreover, the mixture was rinsed with DI water. The residue was subsequently centrifuged to isolate the NC.

#### 2.2.3. Fabrication of Aerogels

**Figure 1:**
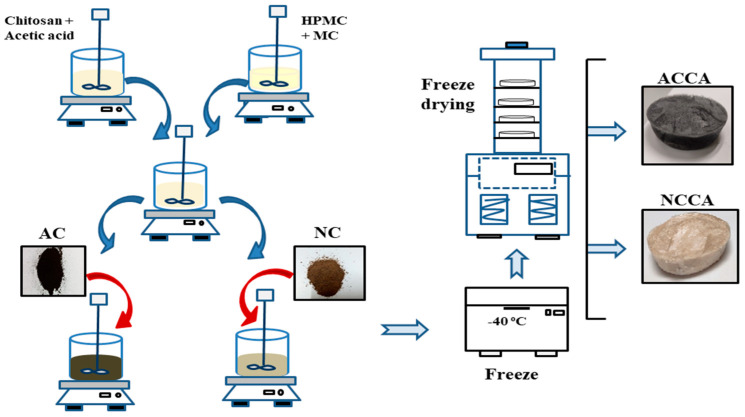
Schematic diagram for the fabrication of activated carbon-chitosan aerogel (ACCA) and nanocellulose-chitosan aerogel (NCCA).

Two biopolymer-based composite aerogels were fabricated using C, NC, and AC as the primary constituents. The precursors gels for the aerogels were generated through a sol–gel technique involving the production of a colloidal solution via the hydrolysis of raw materials, followed by the condensation of sol particles [21]. A schematic diagram of the complete procedure for aerogel preparation is presented in Figure 1. The preparation of the NC/C/HPMC/MC composite aerogel was partially adapted from previous work [22]. Briefly, a chitosan solution was prepared by dissolving 2 g of chitosan powder in 100 mL of 1% acetic acid solution. This was achieved using a magnetic stirrer at room temperature for 1 h. Chitosan, when dissolved in an aqueous solution of CH_3_COOH (pH < 6), acts as a polycation with –NH_3_ groups linked to its backbone. Two positively charged –NH_3_ groups from one or more C polycation chains create two ionic bonds, occurring at numerous sites along the C polycation chains. This process of ionic bond formation leads to the transformation of the C mixture into a water-insoluble hydrogel. Consequently, an appropriate amount of NC was added to the chitosan solution and agitated until a homogenous mixture of NC–C was obtained. At the same time, 0.5 g of HPMC was dissolved in 80 mL of hot water (80 °C) in a 250 mL beaker until completely dissolved. Subsequently, 0.5 g of MC was added to this solution, and the final volume was adjusted to 100 mL. To make a polymeric chain, pre-dissolved HPMC and MC were thoroughly mixed and homogenized with the polycationic chitosan solution. The NC/C/HPMC/MC mixture ratio was adjusted to 5:2:1/2:1/2. The mixture was cooled by placing the beaker in an ice bath. Later, the HPMC–MC solution was added to the mixture of NC–C and agitated at 1000 rpm until a homogeneous composite was achieved. High-speed agitation was utilized to homogenize the NC over the biopolymer mixtures. The pH of the gel-forming solution was kept between 5 and 5.5 [23], measured using a Mettler Toledo pH meter (Mettler-Toledo, Greifensee, Switzerland). The freeze–thaw method was used in three cycles to obtain a stable hydrogel with enhanced association and strength of the gel network. Because of the presence of hydrogen bonds within the hydrogel structure, the hydrogels became spongier and more porous, with a rubber-like texture and highly elasticity [24]. The straightforward blending of a polycation and polyanion results in the creation of hydrogels through intricate coacervation [8]. Because they are oppositely charged, these polymers bind together and form soluble and insoluble blends, which vary based on the concentration and pH of the solutions [23]. While forming composites, the ether groups within the biopolymer film tend to interact with hydroxyl groups through hydrogen bonding [25]. Intermolecular hydrogen bonds facilitate physical cross-linking among biopolymers, leading to the creation of stable three-dimensional structures. Consequently, the resulting aerogels tend to be mesoporous and anisotropic. This observation can be elucidated by forming covalent bonds primarily between carboxylic acid and chitosan, resulting in esters and/or amide groups [26].

A second composite aerogel was made of AC/C/HPMC/MC using the same method but replacing AC with NC. Both the composite aerogels were named: NCCA and ACCA, respectively.

#### 2.2.4. Drying of the Aerogels

The hydrogel obtained was converted into aerogel through a 48 h freeze-drying process using a laboratory-scale Martin Christ freeze dryer Delta 2-24 LSC plus (Christ, Osterode am Harz, Germany). To ensure only sublimation occurred, the suspensions were frozen at a controlled temperature of −20 °C for 24 h before freeze-drying.

### 2.3. Characterization

The composite aerogels’ surface structure and elemental composition were evaluated using a scanning electron microscope (SEM) and energy dispersive X-ray (EDX) analysis with a TESCAN model (MIRA, Brno, Czech Republic) operated at 5 kV. The samples’ surface areas (SA) underwent analysis using N_2_ adsorption at 77 K with an ASAP 2460 instrument from Micrometrics, (Norcross, GA, USA) employing the Brunauer–Emmett–Teller (BET) method. Moreover, Fourier transform infrared spectroscopy (Perkin Elmer/FTIR Spectrum GX, Waltham, MA, USA) was employed to analyze the functional groups found in the aerogel samples across the wavenumber range of 4000–400 cm^−1^. The thermal stability of the composite aerogels was investigated using thermogravimetric analysis (TGA) with a TGA/DSC3+ instrument from Mettler Toledo, Switzerland.

### 2.4. Mechanical Properties

The mechanical behavior of the aerogel samples was characterized using compression testing by the elastic modulus (E), elongation at break (Emax), and tensile strength (TS), as these parameters are closely linked to the internal structure of the sample. An Instron Universal Testing Machine (UTM) model 4502 (Instron Co., Canton, MA, USA) was utilized. Cylindrical aerogel samples were prepared for testing, measuring approximately 13 mm in height and 52 mm in diameter. The samples were positioned between a pair of stationary plates measuring 110 mm in diameter. The compressive Young’s modulus of the aerogels was assessed with a strain limit set at 80% of the initial height of the aerogels, using a testing speed of 5 mm min^−1^. Likewise, the tensile strength (TS) and elongation at break (EAB%) of the aerogels were determined according to ASTM D 882 [27], using the same instrument by fixing the size of the aerogels (10 mm width, 30 mm length). Each type of aerogel underwent testing in at least three replicates. The tensile strength (TS, kPa) from the maximum load was calculated using Equation (1).

(1) $$TS=\frac{F_{Max}}{A}$$

where *F_Max_* is the maximum force applied to the material during the tensile test and A is the original cross-sectional area of the sample being tested. The ratio between the extension and initial length of each aerogel sample was measured for the elongation at break (Emax, %). The aerogel density denoted as *ρ_a_* (g cm^−3^) is determined by measuring the mass and volume of a cylindrical aerogel sample. Porosity, symbolized as *Φ* (%), is calculated using the following equation:

(2) $$\phi =100\;(1-\frac{\rho_{a}}{\rho_{b}})$$

In the equation, *ρ_b_* stands for the average density of the ingredients utilized in crafting aerogels.

### 2.5. Ethylene Adsorption Experiments

The preliminary experiments involved employing a commercial ethylene gas stream at concentrations exceeding those typically found in fruit/vegetable storage. This approach aimed to establish an accelerated testing procedure for evaluating the ethylene adsorption capacity. In order to make an airtight glass container system (1000 mL) assessing the ethylene scavenging capability, a 10 mm hole was drilled into the screw cap, and airtight sealing was attained with the insertion of a silicone rubber closure. A fixed-size (7 cm × 2 cm) and mass (1 g) of aerogel were individually placed inside the container, and the cap was completely tightened. Furthermore, the cap was sealed using parafilm and Teflon for complete isolation within the bottle. The experiments were conducted at room temperature, starting with an initial ethylene gas concentration of 20 ppm. Five milliliters (5 mL) of gas were extracted from each bottle after every 3 h. The ethylene concentrations were recorded daily until saturation was reached. The change in ethylene concentration was analyzed using an advanced Agilent Technologies gas chromatograph (GC) 7890A (Santa Clara, CA, USA), Scientific equipment center, Mae Fah Luang University, Chiang Rai, Thailand, equipped with a flame ionization detector (FID). The experiments were conducted in triplicate, and the outcomes are presented as the average value ± standard deviation. The quantity of ethylene absorbed by the aerogel samples was determined by subtracting the initial quantity of ethylene from the remaining amount within the jar. This indicates the maximum amount of ethylene mass that one unit of aerogel can adsorb in a pure ethylene environment.

The amount of ethylene adsorbed (*q_e_*) was quantified as the volume removed per unit mass of adsorbent material (mL·g^−1^) and was determined from the experimental data in each sample using Equation (3).

(3) $$q_{e}=\frac{\left(C_{i}-C_{f}\right)}{m}\times v$$

Here, *V* represents the available volume for adsorption in the jar (L), *C_i_* and *C_f_* represent the initial and final concentrations of ethylene in the jar (µL L^−1^), and *m* represents the mass of the aerogel (g).

### 2.6. Adsorption Kinetics and Modeling

The adsorption kinetics of ethylene on ACCA and NCCA were investigated to identify the kinetic model that best fits the experimentally obtained data. The pseudo-first-order kinetic model, originally introduced by Lagergren in 1898 for n = 1 (Equation (4)), was initially utilized [28,29]:

(4) $$\frac{dq_{t}}{d_{t}}=K_{1}(q_{e}-q_{t})$$

In this equation, *q_e_* and *q_t_* (mL kg^−1^) denote the amounts of ethylene eliminated per unit mass of the scavenger at equilibrium and at time t (h), respectively. Furthermore, *k* (h^−1^) signifies the rate constant of the pseudo-first-order kinetic model. Integrating Equation (4) yields Equation (5).

(5) $$\ln(q_{e}-q_{t})=\ln q_{e}-kt$$

The non-linearized form of the equation above is provided by Equation (6):

(6) $$q_{t}=q_{e}(1-e^{-kt})$$

The experimental data underwent fitting with Equation (6) to calculate the model parameters (*q_e_* and *k*) using the least squares method. The optimal model parameters were determined by minimizing the sum of the squares of residuals between the observed and predicted values.

Elovich’s equation represents an alternative rate equation derived from adsorption capacity. In 1934, Zeldowitsch’s seminal work established the kinetic law of chemisorption, where the rate of adsorption of CO on MnO_2_ was shown to decrease exponentially as the amount of gas adsorbed increased [30]. Subsequently, this equation has commonly been referred to as the Elovich equation in the ensuing years.

(7) $$\frac{dq}{dt}=ae^{-\alpha q}$$

In the given equation, *α* denotes the initial adsorption rate, and *a* represents the desorption constant within a particular experiment. In simplifying Elovich’s equation, in 1980, Chien and Clayton assumed that *aαt* >> 1, and by incorporating the boundary conditions of *q* = 0 at *t* = 0 and *q* = *q* at *t* = *t*, Equation (7) is transformed into Equation (8).

(8) q=αln(aα)+αln(t)

Accordingly, the constants can be determined from the slope and intercept of a linear plot of *q* against ln(*t*). This Elovich equation is frequently employed to elucidate the kinetics of gas chemisorption onto heterogeneous solids. However, its application is limited, as it describes a limiting property attained by the kinetic curve over time.

As an alternative to Elovich, Ritchie proposed a model for the adsorption of gaseous systems in 1977 [31]. This model makes certain assumptions: in the equation, *n* and *θ* signify the number and proportion of surface sites occupied by an adsorbed gas. Equation (9) is derived with the assumption that the adsorption rate relies solely on the proportion of vacant sites at the given time t.

(9) $$\frac{d\theta }{dt}=\alpha {\left(1+\theta \right)}^{n}$$

Equation (9) integrates to yield Equation (10),

(10) $$\frac{1}{{\left(1-\theta \right)}^{n-1}}=(n-1)\alpha t+1\;for\;n\neq 1$$

or

(11) $$\theta =1-e^{-\alpha t}\;for\;n=1$$

The assumption is made that no site is occupied at *t* = 0. Equation (10) becomes

(12) $$\frac{{q_{\infty }}^{n-1}}{{\left(q_{\infty }-q\right)}^{n-1}}=(n-1)\alpha t+1$$

Similarly, Equation (12) becomes

(13) $$q=q_{\infty }(1-e^{-\alpha t})$$

Here, *q_∞_* represents the amount of adsorption achieved after an indefinite period.

## 3. Results and Discussion

### 3.1. Mechanism for the Preparation of Aerogels

The MC effectively eliminated delamination during the process of homogenization and prevented the aggregation of nanocrystals in the pre-synthesized cellulose dispersion, leading to a uniform NC dispersion in the mixture. The AC and NC obtained from the MNSs were used as adsorbent materials to further enhance the properties of the aerogels for efficient ethylene removal.

### 3.2. Ethylene Adsorption

#### 3.2.1. Adsorption Capacity

**Figure 2:**
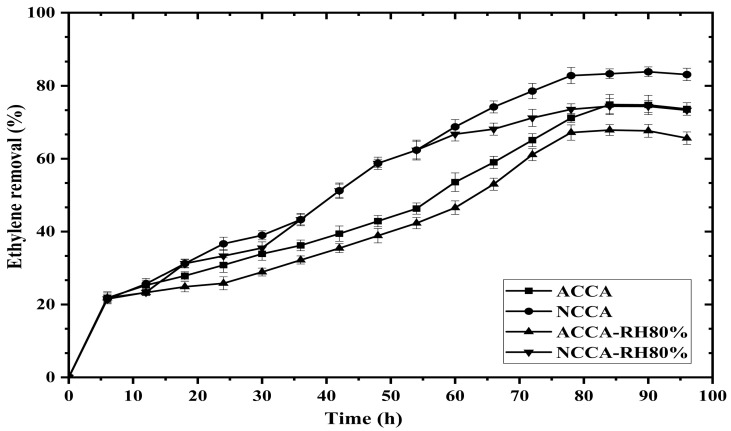
Removal of ethylene with time, *t* (h).

In batch experiments, the capacity (*q_e_*) and efficiency (*ƞ*) of ACCA and NCCA were calculated from the amount of ethylene adsorbed and the ethylene retained in the headspace after the complete adsorption cycle. Furthermore, the adsorption efficiency and adsorbent capacities of ACCA and NCCA at 80% humidity level inside the jars were analyzed, and the data are presented in Figure 2. From Figure 2, the maximum efficiency of NCCA at equilibrium is the highest (83.8%) at 0% humidity, which dropped to 74.4% at 80% humidity. The efficiency of ACCA was 74.8% at 0% humidity, which dropped to 67.8 at 80% humidity. Consequently, the capacity of the scavengers (*q_max_*) was analyzed based on the amount of ethylene adsorbed and is calculated to be 14.97 and 16.77 mL kg^−1^ for ACCA and NCCA, respectively. In contrast, the *q_max_* of ACCA and NCCA at RH 80% dropped to 13.57 and 14.88 mL kg^−1^, respectively. These findings are elucidated by examining the functional groups of the aerogels, which comprise numerous hydroxyl groups on their surface, rendering the material’s tail more hydrophilic. However, more water molecules were entrapped when using ACCA because there are more available surfaces for adsorption, and during competitive adsorption, the moisture contents occupied the available sites for ethylene. In contrast, the decrease in the ethylene removal was not high when using NCCA due to the tightly bound nanofibers, which made micelle inside the composite aerogel that ultimately prevented water molecules from penetrating. Hence, when developing packaging solutions for fresh produce and other moisture-sensitive foods susceptible to ethylene, it is advisable to utilize an ethylene scavenger. This recommendation aligns with findings reported by [32], in which it was demonstrated that halloysite nanotubes designed specifically for scavenging ethylene exhibit an oxygen-adsorption capacity of up to 0.75 wt%. The efficient results in terms of ethylene removal proved the aerogel to be the best scavenger for future use, while, comparatively, the use of NC also reduced the moisture attraction toward the aerogels. To further extend the study, the ethylene removal was assessed with time, and the data were used to predict the best kinetic model.

#### 3.2.2. Adsorption Kinetics

**Figure 3:**
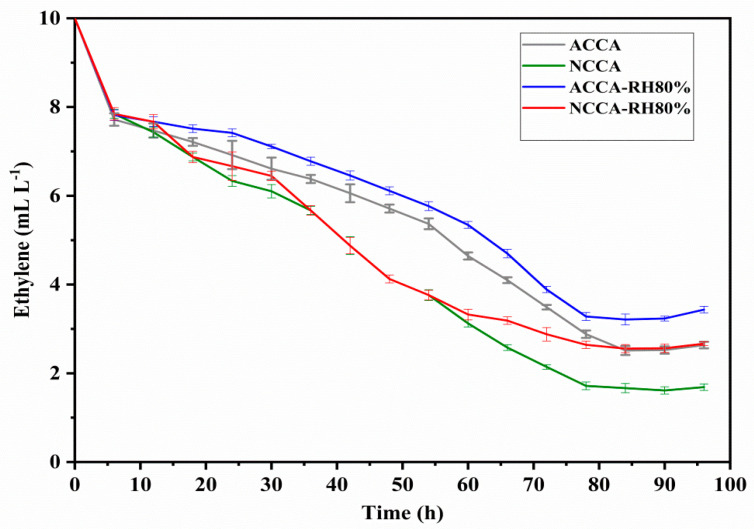
Evolution of the ethylene concentration in the gas phase over time *t* (h).

The removal of ethylene from a batch container is a continuously evolving process; consequently, a study on adsorption kinetics was conducted over a contact time span ranging from 0 to 96 h (Figure 3). The initial ethylene concentration was held constant at 10 mL L^−1^ for 1 g of ACCA and NCCA by keeping a constant relative humidity (RH) of 0% and 80% inside the container at ambient temperature. From Figure 3, the removal was not abrupt. Rather, the trend line was not very smooth for both ACCA and NCCA. Initially, the adsorption was very rapid up to 8 h; afterward, it became gradual but with inconsistent behavior since the high porosity sometimes did not trap the gas molecules properly, especially in the case of NCCA. However, the maximum removal was achieved after 85 h of contact time, which was lower (78 h) in the case of the container with 80% RH. The ethylene present in the moisture-containing container had to compete with water molecules, which also sometimes cover the surface of the scavenger, leading to fewer ethylene molecules going inside. The concentration over time shows both acceleration and deceleration in its decrease due to the larger pore volume of the aerogels allowing the gas molecules to escape. After a certain time, desorption of the ethylene gas occurred once the equilibrium was achieved. The desorption of ethylene with ACCA was higher when compared with NCCA. The obtained results were applied in kinetics isotherm models.

### 3.3. Characterization of the Materials

The three-step process to convert biomass into aerogel caused significant changes in structure and morphology. Characterization was conducted to assess the chemical and morphological structure of the nanocellulose, charcoal, and activated carbon after modification.

#### 3.3.1. BET Surface Area (S_BET_)

Initially, the BET surface area and pore volume of the AC and NC were calculated to be 542.5 ± 11.8 and 325 ± 16.2 m^2^·g^−1^, and 0.7652 ± 0.022 and 1.28 ± 0.064 cm^3^·g^−1^, respectively. Furthermore, the surface area and pore volume of the aerogels were also analyzed to determine the effect of cross-linking AC and NC with the biopolymers. The results for the BET specific surface area of the aerogels are presented in Table 1. The data obtained showed that the specific surface area and pore volume of ACCA and NCCA are 496.2 ± 32.0 m^2^·g^−1^ and 287.6 ± 27.0 m^2^·g^−1^, and 2.78 ± 0.7 cm^3^·g^−1^ and 1.58 ± 0.1 cm^3^·g^−1^, respectively. These values fall within the range reported in previous studies for freeze-dried aerogels [33,34]. The aerogels exhibited a prevalence of micropores and mesopores with a measuring size of less than 50 nm, consequently leading to a notably high specific surface area. The pore volume influenced the amount of gas that can be adsorbed within the aerogel structure. Overall, both the surface area and pore volume positively impacted ethylene gas removal by providing more active sites and space for adsorption within the aerogel structure. Therefore, aerogels with larger surface areas and pore volumes, made from chitosan and nanocellulose or chitosan and activated carbon composites, are generally more effective for ethylene gas removal applications. Once cross-linked with the biopolymers, NC and AC, with their high surface area and abundant hydroxyl groups along their chains [17,35], further enhance the overall performance of the composite aerogels. Their nanostructured morphology offers additional sites for ethylene gas adsorption and facilitates diffusion within the aerogel structure. The aerogels containing AC exhibited the shortest gelation time, smallest chain diameter, and largest specific surface area, attributed to the physical activation of carbon. Conversely, the aerogels synthesized with NC displayed polymer chains characterized by larger diameters, reducing the resulting aerogels’ specific surface area and pore size [35]. However, the porous structure of both AC and NC contributed to the overall pore volume of the composite aerogels, enhancing the capacity for ethylene gas adsorption.

#### 3.3.2. Morphology of the Aerogels

**Figure 4:**
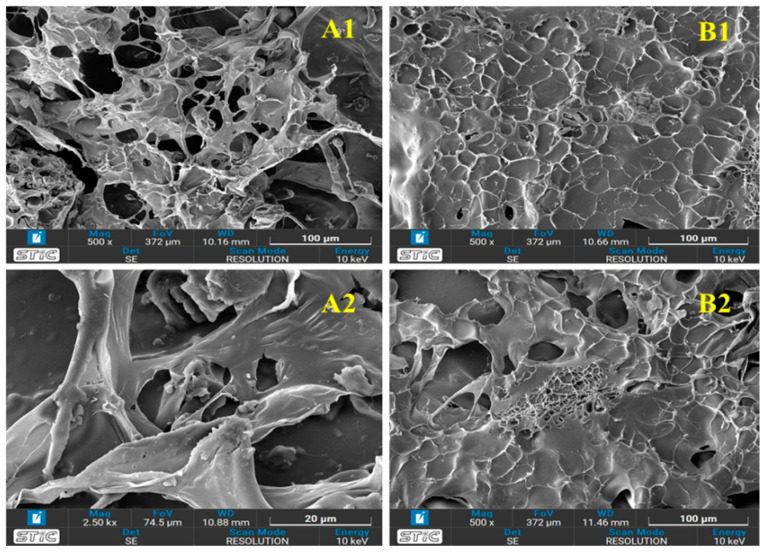
Surface morphology of ACCA (**A1**,**A2**) before ethylene adsorption, and NCCA (**B1**,**B2**) after ethylene adsorption.

Surface morphology and elemental analysis of the aerogels were conducted utilizing SEM micrographs and EDS-mapping techniques. The prepared aerogels’ visual appearance and SEM micrographs are depicted in Figure 4. As is evident in Figure 4, highly porous network structures with macropores were observed in the aerogels prepared in this study, similar to cellulose and activated carbon aerogels reported in earlier studies [17,35]. The aerogel derived from ACCA showcased a uniform, porous, three-dimensional interconnected network structure, created through the self-assembly of cellulose molecules (see Figure 4(A1)). A SEM analysis of composite aerogels containing C, NC, MC, and HPMC would provide insights into the multiscale microstructure of the material, highlighting the interactions between individual components and their contribution to the overall morphology and properties of the composite (Figure 4(B1,B2)). This cellulose aerogel comprised interconnected fibrils made from a biopolymer group. Mixing with activated carbon, it became evident that the porous 3D network of biopolymers remained intact (Figure 4(A2)). However, in comparison to the ACCA, the structure of the NCCA contracted inwardly, causing the pore structure to condense, resulting in smaller pore diameters. A highly porous structure with strong fiber strands exhibits the successful bonding of biopolymers and organic binders. Additionally, it is apparent that NCCA exhibits a smooth surface and formed random connections after pretreatment (Figure 4(B1)). Subsequently, it becomes evident that cellulose fibers are physically interlinked with C, M, and HPMC to form the aerogel structure (Figure 4(B2)).

#### 3.3.3. FTIR Study

The infrared spectra of the aerogels were obtained both before and after exposure to ethylene to comprehend the adsorption process of ethylene on the composite aerogels. A shift of the IR peaks is observed, especially in the range of 2800 to 3500 cm^−1^. In summary, the peaks detected at 1651 and 1555 cm^−1^ are associated with the vibrations of amide I (C–O stretching) and amide II (N–H bending), respectively [17,36]. The peaks within the range of 1300–1400 cm^−1^ correspond to the bending of the OH group’s characteristic of phenol groups present in ACCA. The peaks denote the stretching of CO– bonds in aromatic esters of the cross-link bridges, suggesting the effective cross-linking of the biopolymers [37].

**Figure 5:**
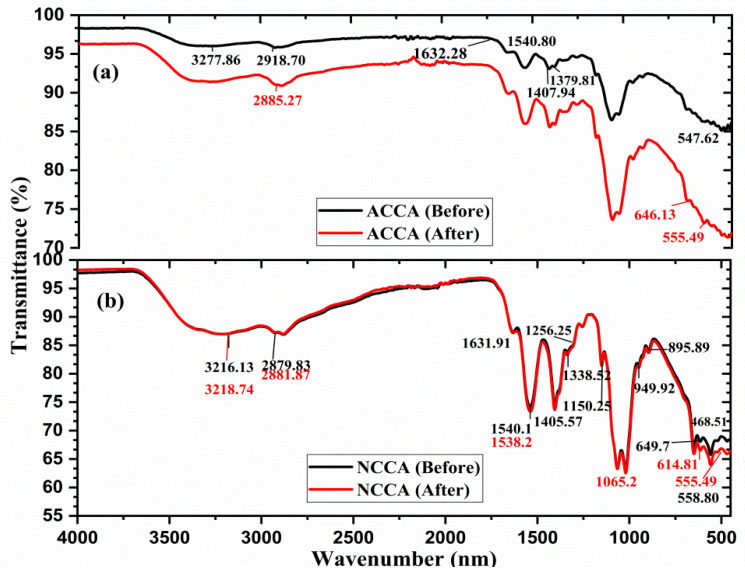
FTIR spectra of ACCA (**a**) and NCCA (**b**) before and after the ethylene adsorption.

Moreover, as depicted in Figure 5b, the wide band observed at 3500–3200 cm^−1^ signifies the stretching of –OH groups present in cellulose, chitosan, methylcellulose (MC), and hydroxypropyl methylcellulose (HPMC) within the NCCA composite aerogel, as reported by [37]. The peak around 2900 cm^−1^ corresponds to the stretching of asymmetric and symmetric –CH_2_ groups [38]. The peak observed around 1730 cm^−1^ is attributed to the stretching of C–O and CO– bonds, characteristic of the carbonyl band of cross-linked ester bonds, as noted by [38]. This implies that chitosan and HPMC cross-link the cellulose chains of MC through ester bonds. The presence of a peak at approximately 1595 cm^−1^ signifies the asymmetric axial deformation of the –COO^−^ anion, suggesting effective cross-linkages facilitated by HPMC and MC. The peaks observed at approximately 1256 cm^−1^ and 1065 cm^−1^ correspond to CO stretching and COC stretching, respectively. These peaks are characteristic of the ether groups within cellulose (glucose units) and those resulting from cross-linking [39,40]. The bands observed at 1150, 1065, and 895 cm^−1^ originate from the saccharide region of chitosan, as documented by [36]. The band observed between 830 and 900 cm^−1^ is associated with the stretching of C–OC– bonds in β-glycosidic linkages connecting glucose units of cellulose, HPMC, and MC. Moreover, the subtle peaks detected in NCCA between 1050 and 1130 cm^−1^ are indicative of CO–stretching in alcohol, phenol, carboxyl, ether, and ester groups.

The IR spectra of ACCA and NCCA given in Figure 5b show that some new peaks appeared after the ethylene adsorption and some old peaks disappeared. The ATR-FTIR spectra of the ACCA when exposed to ethylene reveal distinct peaks at 1380 cm^−1^, indicating the presence of π-adsorbed ethylene [41]. Ethylene can be adsorbed within the surface pores through two types of intermolecular interactions: cation···π and C–H···O interactions. The surface of activated carbon provides abundant oxygen atoms serving as adsorption sites, enabling the formation of C–H···O interactions between the C–H groups of ethylene and the electronegative oxygen of activated carbon [41]. Similarly, the cation···π interaction may occur between the π-orbital of ethylene and the cation within activated carbon. However, both the cation···π and C–H···O interactions are considered relatively weak hydrogen bonds that exhibit reversibility [42].

Examining the ATR-FTIR spectra of the NCCA sample upon exposure to ethylene reveals peaks at 1338 cm^−1^, indicative of π-adsorbed ethylene. Furthermore, a minor band at 1406 cm^−1^ corresponds to di-σ adsorbed ethylene on silica. Additionally, bands at 1872 cm^−1^ originate from the C–C stretching vibration [37]. Significantly, the lack of these peaks in the scavenger serves as confirmation of ethylene adsorption onto the NCCA. The presence of an abundant active hydroxyl group on the surfaces of NC and AC enhances the formation of intramolecular and intermolecular hydrogen bonds among NC, AC, and the biopolymer inside the framework structure [30].

In summary, the peaks observed in both the ACCA and NCCA spectra at 468, 555, 646, 615, and 895 cm^−1^ indicate the distortion of Si–O–Si and Al–O–Si bonds, along with the presence of O–H and internal hydroxyl groups [43,44].

#### 3.3.4. Thermogravimetric Analysis (TGA)

**Figure 6:**
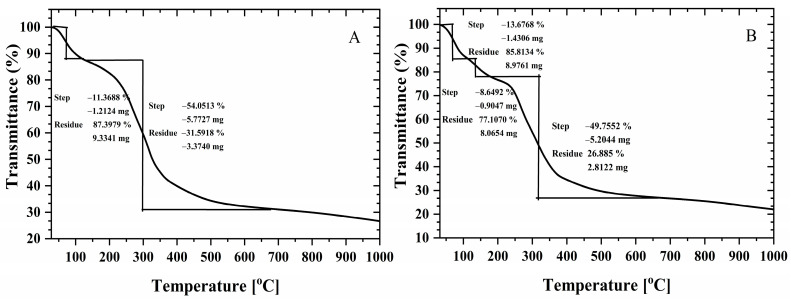
TGA of ACCA (**A**) and NCCA (**B**).

The thermal stability of both the ACCA and NCCA aerogels was assessed through TGA/DSC analysis, conducted from room temperature (RT) to 1000 °C at a heating rate of 5 °C min^−1^. The weight % versus temperature curves depicted in Figure 6 demonstrate a similar pattern, revealing that initial weight loss occurred between 60 and 64 °C for both aerogels, attributable to the removal of residual water molecules from the specimens. The percentage loss due to the moisture content was recorded as 11.37 and 13.68% for ACCA and NCCA, respectively. Subsequently, a distinct and rapid weight reduction commenced around 152 °C and persisted until approximately 650 °C for ACCA, resulting in a weight loss of 54.05%. In contrast, for NCCA, the sharp weight loss occurred in two-step thermal degradation, between 108 and 174 °C and between 217 and 612 °C, with weight losses of 8.6 and 49.7%, respectively. These patterns can be ascribed to the thermal decomposition of organic components present in the specimens and are also due to the decomposition of oxygen-containing functional groups and the evaporation of the entrapped solvent [45]. The thermal decomposition of both samples was negligible beyond 650 °C, with a weight loss of approximately 68% for ACCA and 73% for NCCA, respectively, when the temperature reached 800 °C. This difference in weight loss could be attributed to variations in the rate of organic matter removal. These organic residues arise as by-products from the reaction of biopolymers in the presence of additional compounds within the precursor during the stages of hydrolysis and solvent condensation, occurring throughout the cross-linking and drying processes [46]. Notably, weight reduction occurred between 240 °C and 560 °C for the cross-linked aerogels, corresponding to depolymerization, polymer chain fragmentation, and cross-linkage disruption [46]. In the latter portion of the TG curve, it is evident that the NCCA aerogels underwent greater thermal decomposition than ACCA. This can be attributed to the cross-linking process of the aerogels, where hydrogen bonds within the molecular chains were reformed or reconnected. This subsequently formed a more organized network structure involving activated carbon and organic bonding agents, such as HPMC and MC. In essence, the formation of cross-linkages contributed to enhancing the thermal stability of the aerogels [47].

#### 3.3.5. EDS-Mapping

**Figure 7:**
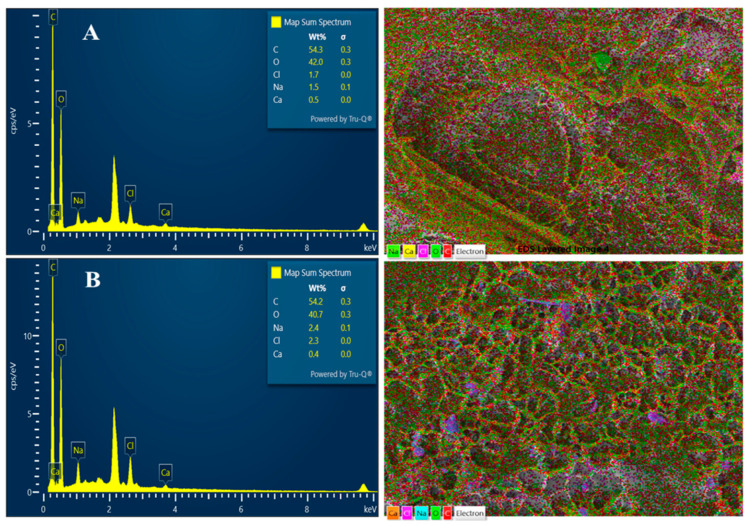
EDS Mapping of ACCA (**A**) and NCCA (**B**) after ethylene adsorption.

Figure 7A,B shows the elements and the map of ACCA and NCCA after the ethylene adsorption. EDS mapping provides visual insights into how ethylene molecules interact with and are adsorbed by the two different aerogel compositions, offering valuable information for understanding their adsorption mechanisms and efficiency. The EDS analysis detected the presence of silicon, aluminum, oxygen, and sodium in all the samples, which is visually represented by distinct colors in the map (Figure 7A,B). The EDS mapping of both aerogels also shows the distribution of elements within the aerogels after ethylene adsorption. The key elements of interest would include carbon (from activated carbon), oxygen (from C/HPMC/MC and NC), and potentially hydrogen (from ethylene). The mapping reveals the spatial arrangement and concentration of these elements, indicating where ethylene molecules are adsorbed within the aerogel structure. Variations in the distribution and concentration of these elements between NCCA and ACCA highlights differences in their ethylene adsorption capabilities.

### 3.4. Mechanical and Physical Properties

The mechanical and physical properties of the aerogel samples are listed in Table 1. From the table, it can be seen that the porosity and the pore volume of the aerogels are very high; inversely, their tensile strength and elongation are not high. The porosity of both aerogels is very high due to their dehydration and the removal of bound water from their inner structure, which ultimately increased the pore volume of both aerogels. In comparison, the pore volume and porosity of ACCA are slightly higher than NCCA due to the addition of activated carbon. The density of ACCA and NCCA is calculated to be 0.092 and 0.077 g cm^−3^, respectively. A similar result was obtained by [9]: their obtained carbon aerogels had extremely low density (0.075–0.174 g cm^−3^). To confirm the results and avoid human errors, the density measurement of the aerogels was repeated by a pycnometer [14]. The results were recorded as 0.098 g cm^−3^ (ACCA) and 0.080 g cm^−3^ (NCCA). The low density indicates that aerogels can be the best materials for insulation and adsorption purposes.

A tensile strength test of the aerogels was conducted to indicate the maximum stress reached by the composites, and their tensile elongation at break represents the stretching capacity of the aerogels [48]. The tensile strength of ACCA was found to be 48.2 ± 1.7 kPa, and that of NCCA was found to be 36 ± 1.1 kPa. Generally, aerogels depict lower tensile strength as compared to film-based food packaging material. The reduced tensile strength in the aerogels could be attributed to the alterations in their properties following dehydration. This decline in mechanical strength is often associated with factors such as fiber diameter and porosity, wherein a larger diameter and higher porosity tend to correspond to lower mechanical strength [49]. A similar mechanism of the tensile strength of aerogel formulation from nanocellulose was also reported in a previous work [50]. During drying, water was removed from the cellulose fibers, facilitating the formation of hydrogen bonds between adjacent fibers and resulting in the development of a dense fiber network. This irreversible process, referred to as “hornification”, enhances the strength of both ACCA and NCCA, rendering them durable [50]. Nevertheless, this level of tensile strength is deemed sufficient for chitosan-based aerogels, as it enables them to endure the challenges of transportation and handling without compromising the integrity of the packaged contents. However, depending on the end application, the mechanical properties of the aerogels can further be improved by fluctuating the ratio of the bonding biopolymers in the mixture. Overall, the mechanical and physical properties of ACCA and NCCA are highly supportive to let them become efficient materials to be used in various applications, from food packaging to building insulations.

## 4. Adsorption Modeling

**Figure 8:**
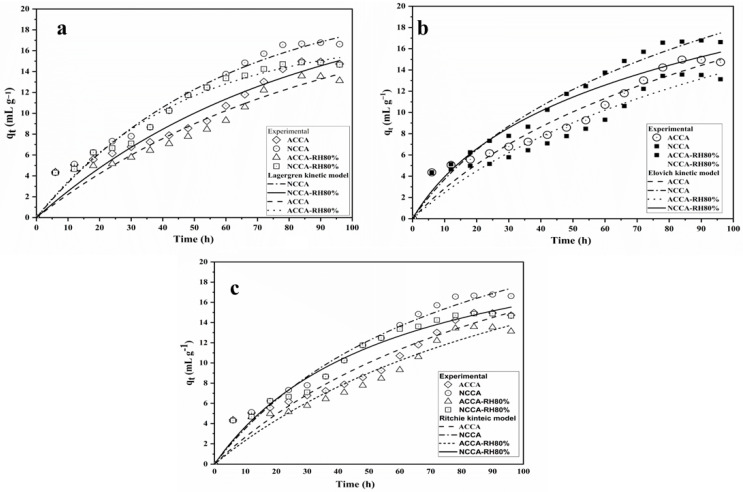
Lagergren kinetic model (**a**), Elovich model (**b**), and Ritchie’s model (**c**).

Adsorption isotherms play a crucial role in explaining the rate at which ethylene molecules adsorbed to the surface of the aerogels. This is essential for comprehending the dynamics of the adsorption kinetic process. To define this kinetics process, Lagergren pseudo-second-order kinetics was studied for the ACCA and NCCA at different RH (Figure 8a). The coefficient of determination (R^2^) serves as an indicator of the degree of fitting of the model to the experimental data points. Table 2 lists the regression coefficients and adsorption capacities for both aerogels with and without RH. The experimental data clearly adhered to the Lagergren pseudo-first-order kinetic model at 0% relative humidity (RH), with R^2^ values of 0.97% (ACCA) and 0.98% (NCCA), respectively. However, at 80% RH, the R^2^ values of 0.92 (ACCA) and 0.95 (NCCA) suggest a minor deviation of the experimental data points from the predicted model. This deviation could be attributed to the competition between water molecules and ethylene molecules competing for the same adsorption sites of the ethylene scavenger [51]. When exposed to 80% humidity, the scavenger might have rapidly interacted with water molecules, potentially impacting ethylene adsorption. Furthermore, the chance of water vapor condensation at 80% humidity cannot be overlooked. These elements may have led to the decrease in model fitting (R^2^ = 0.92) for ACCA at 80% RH. A better fit of the model was obtained for NCCA at all the RH conditions. The overall results indicate that the *q_e_* values were more consistent using NCCA, where *q_e_* indicates moderate removal of ethylene at equilibrium of the scavengers. The exponential Elovich equation finds broad utility in describing chemisorption kinetics and has been employed to characterize the kinetics of heterogeneous exchange reactions. Moreover, it has a longstanding history of application in explaining the kinetics of gas adsorption on solid surfaces. The results of the Elovich model are very similar to that of the Lagergren model (Figure 8b). However, an increase in the R^2^ value of NCCA explains well the involvement of intraparticle diffusion within the scavenger.

In 1977, Ritchie introduced a model for the adsorption of gaseous systems [31]. Ritchie’s pseudo-second-order kinetics explain the complete adsorption cycle for a longer adsorption process. The current experimental findings are consistent with Ritchie’s straightforward theory, which proposes that the rate of gas adsorption on a solid is directly proportional to the fraction of unoccupied sites raised to a certain power. The calculated coefficient of regression (R^2^) for ACCA and NCCA under this theory is in the range of (0.95 to 0.98), respectively. The scavenger capacities (*q_e_*) of ACCA and NCCA through model analysis are 14.4 to 15.7, respectively. However, the R^2^ and *q_e_* values at 80% RH are similar to those of the Lagergren kinetics model. Consequently, the plots give linear lines; however, the intercepts at the onset of experiments are notably high (Figure 8c). In certain instances, this could be attributed to experimental variables impacting the determination of the initial time. For instance, the moment when gas is introduced to the sample may not represent the actual starting point, as time is required for the gas to stabilize pressure within the reaction vessel. Alternatively, it might result from rapid reactions occurring initially on the specific favored surface sites [31]. However, it appears that the majority of adsorption adheres to the kinetic equations outlined in Ritchie’s model. This phenomenon eliminates the fluctuations in gradients observed in the Elovich plots, which were previously interpreted as indicative of alterations in the reaction mechanism. Therefore, it is suggested that these fluctuations arise from the structure of the Elovich equation and do not reflect an actual physical phenomenon.

## 5. Conclusions

A unique method was employed to produce aerogels by combining various biopolymers with nanocellulose and activated carbon sourced from macadamia nutshell. The wide band observed at 3500–3200 cm^−1^ signifies the stretching of –OH groups present in cellulose, chitosan, methylcellulose (MC), and hydroxypropyl methylcellulose (HPMC) within the ACCA and NCCA composite aerogels, whereas, the ATR-FTIR spectra of the ACCA and NCCA exposed to ethylene reveal distinct peaks at 1380 cm^−1^ and 1338 cm^−1^, indicating the presence of π-adsorbed ethylene. The combined effect of their surface area and pore volume increases the availability of active sites for ethylene gas adsorption and creates ample space for gas molecules within the aerogel structure. The ethylene removal and adsorption capacity were found to be 83.86% and 16.77 mL·kg^−1^ for NCCA, and 74.75% and 14.97 mL·kg^−1^, for ACCA, respectively. However, under 80% RH, the ethylene removal rates dropped to 74.43% (NCCA) and 67.86% (ACCA). Lagergren’s pseudo-second-order kinetic model was the best fit for both NCCA and ACCA, with R^2^ of 0.97 and 0.98, respectively. Furthermore, the superior fit of Ritchie’s pseudo-second-order kinetics compared to experimental values suggests a complete adsorption cycle for longer adsorption processes. The calculated coefficient of regression R^2^ for NCCA and ACCA under this model ranged from 0.95 to 0.98, respectively. These results affirm that the ability to combine NC- and AC-based composite aerogels with other biopolymers offers opportunities to customize the properties of resulting hybrid materials to meet specific sustainable packaging needs. Additionally, they can assist in prolonging the shelf life of packaged products by inhibiting microbial growth. The precursors used in the preparation of the aerogels are all environmentally friendly, biodegradable, and can be recycled into other useful products. However, the composition of the aerogels’ ingredients can be altered depending on the intended application to modify their physical and chemical structure.

## Figures and Tables

**Table 1:** Physical and mechanical properties of ACCA and NCCA aerogels.

Parameter	ACCA	NCCA
BET specific surface area (m^2^·g^−1^)	496.2 ± 32.0	287.6 ± 27.0
Pore volume (m^3^·g^−1^)	2.78 ± 0.70	1.58 ± 0.13
Pore size (μm)	3.41 ± 0.06	2.82 ± 0.03
Porosity *Φ*, (%)	96.10 ± 0.60	95.00 ± 0.84
Density (g cm^−3^)	0.094 ± 0.001	0.077 ± 0.004
Max force (N)	84.97 ± 0.72	53.97 ± 1.04
Compressive modulus (kPa)	48.20 ± 0.94	36.10 ± 0.76
Elongation at break, EB (%)	12.26 ± 0.14	9.46 ± 0.04

**Table 2:** Summary of the kinetic model’s analysis.

Model	Parameters	Ethylene Scavenger			
		ACCA	NCCA	ACCA-RH80%	NCCA-RH80%
Experimental results	*q_e_*	14.973	16.773	13.57	14.88
Lagergren pseudo-first-order kinetics	*q_e_*	14.54	17.23	13.97	15.36
	R^2^	0.97	0.98	0.97	0.97
Elovich	*q_e_*	15.54	16.11	14.41	14.34
	R^2^	0.94	0.97	0.92	0.97
	A	0.3232	0.44	0.275	0.51
	B	0.088	0.089	0.087	0.125
Ritchie’s	*q_e_*	15.72	16.44	12.76	14.41
	R^2^	0.95	0.98	0.91	0.97
	k	0.0027	0.0035	0.0024	0.007

## Data Availability

The original contributions presented in the study are included in the article, further inquiries can be directed to the corresponding authors.
